# Construction of Antibacterial MoS_2_-ACF Phenotype Switcher for Bidirectionally Regulating Inflammation–Proliferation Transition in Wound Healing

**DOI:** 10.3390/ma18050963

**Published:** 2025-02-21

**Authors:** Mengxin Mao, Diyi Li, Yunyun Wu, Bing Li, Xiaoqing Han, Jiao Yan, Lei Shang, Haiyuan Zhang, Xi Li

**Affiliations:** 1 School of Chemistry and Life Science, Changchun University of Technology, Changchun 130012, Chinayywu163@163.com (Y.W.);; 2 Laboratory of Chemical Biology, Changchun Institute of Applied Chemistry, Chinese Academy of Sciences, Changchun 130022, China

**Keywords:** MoS_2_-activated carbon fiber materials, bidirectional regulation, inflammation–proliferation transition, macrophage reprogramming, wound healing

## Abstract

The transition between the inflammatory phase and the proliferative phase is critical for wound healing. However, the development of proper switchers that can regulate this transition is facing great challenges. Macrophages play versatile roles in all wound healing phases because they can readily switch from pro-inflammatory M1 phenotypes to anti-inflammatory M2 phenotypes in response to different microenvironment stimuli. Herein, taking advantage of enhanced electron transfer by coupling MoS_2_ with a highly conductive activated carbon fiber (ACF) network, a MoS_2_-ACF heterojunction structure was constructed as a macrophage M1-M2 phenotype switcher (MAPS) for regulating inflammation–proliferation transition to accelerate wound healing. In the early stages of wound repair, MAPS-mediated photothermal effects with near-infrared laser irradiation could promote macrophage reprogramming to the M1 phenotype, which can expedite inflammation. NIR photo-induced hyperthermia, together with M1 macrophages, directly and indirectly kills bacteria. Later, during the healing process, the MAPS could further reprogram macrophages towards the M2 phenotype via its inherent reactive oxygen species (ROS) scavenging ability to resolve inflammation, promoting cell proliferation. Therefore, MoS_2_-ACF heterojunction structures provide a new strategy to modulate inflammation–proliferation transition by rebalancing the immuno-environmental equilibrium of macrophage M1/M2 phenotypes.

## 1. Introduction

Wound healing is a vital physiological process in maintaining skin integrity after trauma [1]. In 2018, Medicare spent USD 28.1 to 96.8 billion on acute and chronic wound treatment [2]. From an economic point of view, the global wound treatment market is expected to reach USD 15–22 billion by 2024 [3]. The wound healing process is usually characterized as four distinct but overlapping programmed phases: hemostasis, inflammation, proliferation, and remodeling [4]. For a wound to heal successfully, these four phases must occur in an appropriate sequence and time frame [5]. The deregulation of any of these steps may cause impaired healing, e.g., excessive scarring or chronic difficult-to-heal ulcers [6]. Of the whole wound healing process, the transition from the inflammatory phase to the proliferative phase is the most critical step.

Macrophages play multiple roles in the transition from the inflammatory phase to the proliferative phase [4,7]. In the early wound stage, macrophages assume the M1 ‘classically activated’ phenotype by releasing cytokines (e.g., TNF-α, IL-6, IL-12) that promote the inflammatory response by activating and recruiting additional leukocytes to prevent pathogenic bacterial infection [8,9,10]. Later, macrophages are also responsible for inducing and scavenging apoptotic cells (including neutrophils), thus paving the way for a phenotypic transition from the M1 phenotype to an ‘alternatively activated’ M2 phenotype that stimulates keratinocytes, fibroblasts, angiogenesis, the secretion of anti-inflammatory mediators (IL-10), and growth factors (e.g., TGF-β, PDGF, TNF-β) to promote tissue closure [11]. In this way, the transition from the inflammatory phase to the proliferative phase could be orchestrated by the immune macrophage phenotype and its functions. The phenotypic transformation of macrophages is a dynamic and reversible process driven by the factor of extracellular environmental stimuli. Thus, the development of new drugs/material systems capable of properly modulating macrophage activation will be beneficial for the enhancement of wound healing efficiency [12,13,14,15]. Specific ligands mediate these distinct changes via toll-like receptors. Pathogen-associated molecular markers, such as lipopolysaccharide (LPS), damage-associated molecular markers, growth factors, and Th1 cytokine, can elicit pro-inflammatory M1 activation. Furthermore, physical photothermal/photodynamical effects mediated by photosensitizers, such as FeS_2_ [16], Cu_2−x_Se [17], Au@Se [18], bradykinin (BK) aggregation-induced emission nanoparticles [19], thermo-sensitive lipids [20], etc., can also induce the macrophage M1 phenotype. On the other hand, Th2 cytokines (e.g., IL-4, IL-13) [21] and bioactive reactive oxygen species (ROS)-scavenging nanozymes (CeOx [22], Ag [23], MnO_2_ [24], MnFe [25]) can activate alternative M2 responses to promote cell proliferation. However, only single-transition induction has been widely studied to promote the wound healing process, such as controlling inflammation via M1 macrophages or prolonging the proliferative stage via M2 macrophages. The modulating factors that can facilitate the inflammation–proliferation transition have rarely been investigated. In addition, among the whole wound healing process, mechanical stress in tissues triggers immune effects due to the fibroblast production during inflammation and proliferation [26,27]. Thus, the optimal stage for promoting wound healing through immunomodulation is the inflammation and proliferation stage, and the transition from the inflammatory phase to the proliferative phase is the most critical step.

**Scheme 1:**
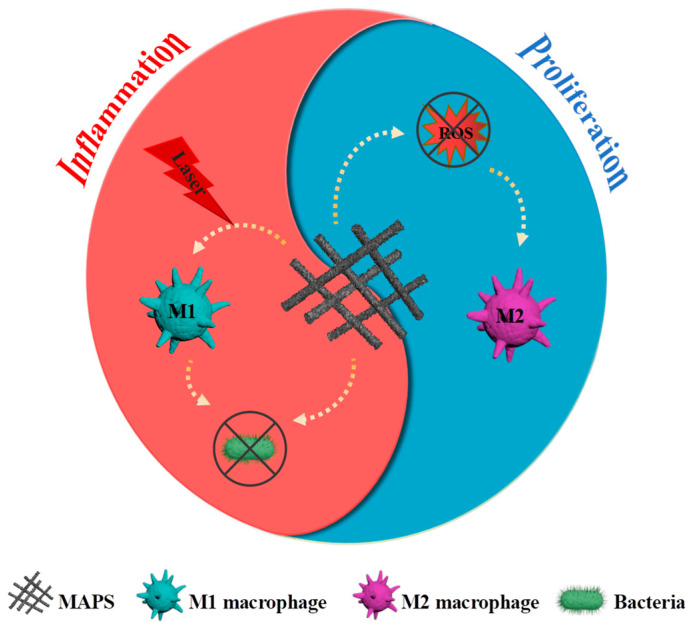
Schematic illustration of MAPS for the regulation of inflammation–proliferation transition to accelerate wound healing.

Inspired by the concept that efficient electron transfer caused by doping molybdenum sulfide (MoS_2_) onto activated carbon fiber (ACF) [28,29] could enhance its photothermal and antioxidative properties [30,31], we designed a MoS_2_-ACF heterojunction structure serving as a macrophage M1-M2 phenotype switcher (MAPS) to adjust the wound healing transition from the inflammatory phase to the proliferative phase (Scheme 1). ACF, a soft and flexible textile fiber, is used commercially to protect wounds from exogenous infection. It provides a structure-like native extracellular matrix, which is an appropriate environment to balance moisture and gas permeability around wound sides [32,33]. Dense MoS_2_ being interconnected with ACF networks provides a rough surface to enhance wound closure. Under near-infrared (NIR) laser irradiation, a MAPS could efficiently inhibit bacterial growth due to the high photothermal conversion efficiency contributed by the coupling of MoS_2_ and ACF. Furthermore, this MAPS-potentiated hyperthermia facilitates the reprogramming of macrophages towards the M1 phenotype, promoting the inflammatory response to prevent bacterial growth. The precise regulation of redox signals is very important for the transition from inflammation to proliferation during wound healing [4]. Subsequently, scavenging residual ROS via antioxidants can initiate proliferation via the reversible stimulation of macrophage phenotypic switching to M2. MoS_2_ is a model antioxidant nanozyme that can vertically align on different substrates simultaneously to effectively lower free radical levels in the wound area and reduce oxidative injury [34,35,36]. However, due to the low inherent electronic conductivity of MoS_2_, electron transfer from Mo^4+^ to Mo^6+^ may be seriously influenced. A MAPS to harmoniously graft MoS_2_ to highly conductive ACF could ensure smooth redox cycling between Mo^4+^ and Mo^6+^ [37]. The enhanced antioxidative properties activate macrophage phenotypic switching from the pro-inflammatory M1 phenotype to the reparative M2 phenotype, thus triggering the inflammation–proliferation transition. A series of assessments in vitro and in vivo have proven the significant performance enhancement of the MAPS for the modulation of the inflammation–proliferation transition.

## 2. Materials and Methods

### 2.1. Materials and Reagents

PAN (Polyacrylonitrile)-based carbon fiber (T300, 3 K, diameter of 7 μm) was received from Toray (Tokyo, Japan) Industries, Inc. Acetone, nitric acid, and hydrogen peroxide were obtained from Xilong Science Co., Ltd. (Shantou, China). Ammonium tetrathiomolybdate ((NH_4_)_2_MoS_4_) was purchased from Shanghai Aladdin Biochemical Technology Co., Ltd. (Shanghai, China). Dimethylformamide (DMF) was purchased from Tianjin Fuyu Fine Chemical Co., Ltd. (Tianjin, China). A superoxide dismutase (SOD) assay kit and catalase (CAT) assay kit were purchased from Jiangsu Keygen Biotech Co., Ltd. (Nanjing, China). Fetal bovine serum (FBS) was purchased from Gibco Life Technologies Co., Ltd. (Waltham, MA, USA). Penicillin, streptomycin, and trypsin-EDTA were purchased from Merck Millipore (Burlington, MA, USA). Dulbecco’s modified Eagle’s medium (DMEM) was obtained from Hyclone (USA). FITC anti-mouse CD80 antibodies and APC anti-mouse CD206 antibodies were obtained from Beijing BioLegend Biotechnology Co., Ltd. (Beijing, China). A cell proliferation test kit (kFluor488-EdU (5-Ethynyl-2′-deoxyuridine)) and a TNF-α and IL-10 ELISA Kit were purchased from Shanghai Beyotime Biotechnology Co., Ltd. (Shanghai, China). Phosphate-buffered saline (PBS), 3-(4,5-dimethylthiazol-2-yl)-2,5-diphenyltetrazo-lium bromide (MTT), and dimethyl sulfoxide (DMSO) were obtained from BBI Life Sciences Co., Ltd. (Shanghai, China). The water used in the experiments was from a Milli-Q water purification system (Millipore, Bedford, MA, USA).

### 2.2. Characterization of Materials

Images of the size and morphology of the products were obtained via a Field-Emission Scanning Electron Microscope (FE-SEM, JEOL JSM-7610F, Tokyo, Japan) with an accelerating voltage of 5 kV. X-ray diffraction (XRD) patterns were obtained with a Smartlab X-ray diffractometer (Rigaku, Tokyo, Japan). Raman spectra data were obtained with a Raman microscope (LABRAM HR Evolution, Paris, France) equipped with a 532 nm excitation laser. X-ray photoelectron spectroscopy (XPS) measurements were taken with an ESCALAB MKII photoelectron spectrometer (Thermo Fisher Scientific, Waltham, MA, USA) with Al as the anode. Zeta potential measurements were carried out on a Nanosizer ZS apparatus (Malvern, Malvern, UK). Ultraviolet–visible–near-infrared spectroscopy (UV-VIS-NIR, Cary 5000, Agilent, Santa Clara, CA, USA) was used for the absorbance measurements. The resistance of carbon fiber was measured with an LCR meter mode-of-precision impedance analyzer test (Wayne Kerr WK 6500B, Bognor Regis, UK). We tested the volume resistivity of the carbon fiber monofilament according to GB/T 32993-2016 [38], and the conductivity value was obtained according to the reciprocal quantity of resistivity.

### 2.3. Preparation of MoS_2_ Particles

**Scheme 2:**
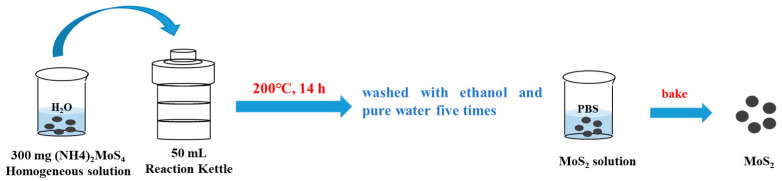
Schematic showing MoS_2_ synthesis.

The synthesis of MoS_2_ particles was revised based on a previously reported hydrothermal protocol [39]: (NH_4_)_2_MoS_4_ (300 mg) was dissolved in pure water (30 mL) by vigorous stirring (500 rpm) to obtain a homogenous solution; then, the solution was transferred into a stainless-steel autoclave (50 mL) lined with polystyrene and maintained at 200 °C for 14 h. The final black product (MoS_2_) was washed with ethanol and pure water five times and then re-dispersed in PBS for further use (Scheme 2).

### 2.4. Preparation of MAPS

**Scheme 3:**
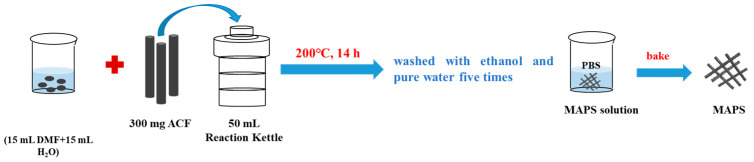
Schematic showing MAPS synthesis.

Firstly, the carbon fiber (CF) was refluxed in acetone solution for 10 h, soaked in concentrated nitric acid for about 4 h, and washed with pure water until the pH of the solution became neutral. Then, activated carbon fiber (ACF) was obtained. The functionalization of ACF with MoS_2_ was carried out via the following methods: (NH_4_)_2_MoS_4_ (150 mg) and ACF (300 mg) were dissolved in a mixture of DMF (15 mL) and pure water (15 mL) by vigorously stirring (500 rpm); then, the solution was transferred into a steel autoclave (50 mL) lined with polystyrene and maintained at 200 °C for 14 h. The black product (MAPS) was washed with ethanol and pure water five times and then re-dispersed in PBS for further use [40] (Scheme 3).

### 2.5. Photothermal Performance Evaluation

Briefly, the PBS solution containing the materials was irradiated under an NIR laser (808 nm). During the experimental process, a high-precision thermocouple probe was inserted vertically into the solution and the temperature was recorded every 30 s for 10 min. To calculate the photothermal conversion efficiency, the laser was removed after 10 min of irradiation and the solution was cooled down naturally. Then, the photothermal conversion efficiency (η) of the material was determined based on the following Equation (1):

(1) $$\eta =\frac{hA\Delta T_{\max}-Q_{s}}{I(1-10^{-A\lambda })}$$

where h is the heat transfer coefficient, A is the surface area of the container, ΔTmax is the temperature change at the maximum steady-state temperature, Qs is the heat associated with the light absorbance of the solvent, I is the laser intensity, and Aλ is the absorbance of samples at 808 nm.

### 2.6. SOD and CAT Enzyme Activity Evaluation

The SOD-like activity of the MAPS was measured with a total superoxide dismutase assay kit with WST-8 (2-(2-Methoxy-4-nitrophenyl)-3-(4-nitrophenyl)-5-(2,4-disulfophenyl)-2H-tetrazolium sodium salt). MAPSs with different concentrations (the content of Mo was 0, 3.9, 7.8, 15.6, 31.3, 62.5, 125, 250, 500, and 1000 μg mL^−1^) were co-incubated with the analytical reagents xanthine and xanthine oxidase for 30 min to generate superoxide radicals from the oxygen. Then, a SpectraMax M3 microplate reader (Molecular Devices, San Francisco, CA, USA) was applied to record the absorbance at 450 nm. The decrease in absorption value means that superoxide radicals were inhibited.

The CAT-like activity of the MAPS was elevated via the catalase activity assay kit. MAPSs with different concentrations (the content of Mo was 0, 3.9, 7.8, 15.6, 31.3, 62.5, 125, 250, 500, and 1000 μg mL^−1^) were incubated with 10 mM H_2_O_2_ for 20 min. A SpectraMax M3 microplate reader was used to record the absorbance at 520 nm and then obtain the concentration of H_2_O_2_ according to the manufacturer’s constructions.

The kinetic measurements were performed in time course mode to calculate the MAPSs’ Michaelis–Menten constant (Km) via the Lineweaver–Burk plot: 1/V0 = (Km/Vmax)/c + 1/Vmax, where V0 is the initial velocity, Vmax is the maximal reaction velocity, and c is the concentration of the substrate [41,42].

### 2.7. Cell Culture

Mouse embryonic fibroblast cell lines (3T3) and monocyte macrophage lines (Raw 264.7) were cultured in Dulbecco’s modified Eagle’s medium (DMEM) containing 10% fetal bovine serum, 100 U mL^−1^ penicillin, and 100 μg mL^−1^ streptomycin. The cells were cultured at 37 °C in a humidified 5% CO_2_ atmosphere. The culture medium was changed every day and the cells were digested and passaged with trypsinization before fusion.

### 2.8. Cell Viability Assay Assessed by MTT

3T3 cells (1 × 10^4^, in 100 µL DMEM) were seeded in 96-well plates for growth overnight; then, 100 µL fresh culture medium containing different concentrations of MAPSs (the content of MoS_2_ was 0, 0.78, 1.56, 3.13, 6.25, 12.5, 25, 50, 100, and 200 µg mL^−1^) was used to replace the old culture medium and cultured for 6 h. It was treated with an NIR laser under different conditions (0, 0.25, 0.5, 0.75, 1.0, 1.5 W cm^−2^; 0, 5, 10, 15 min) and cultured for 18 h. The culture medium was removed and 100 µL of MTT (5 mg mL^−1^) stock solution was added to each well. After 3.5 h, the medium was removed and 150 µL DMSO solution was added to each well; then, the cells were incubated on the shaker for 15 min. Finally, the absorbance at 490 nm was recorded with a SpectraMax M3 microplate reader.

### 2.9. In Vitro Effect of MAPS on Macrophage Phenotype Modulation with NIR Laser Irradiation

Next, 1 × 10^5^ Raw 264.7 cells were seeded in 6-well plates and treated with the MAPS for 6 h. An NIR laser (0.75 W cm^−2^, 10 min) was further applied to stimulate macrophages. LPSs (lipopolysaccharides, 1 μg mL^−1^) were used to stimulate the macrophages for 3 h, characterized by a strong induction of CD80 (M1 macrophage marker) and downregulation of CD206 (M2 macrophage marker), representing typical M1 macrophages. Cells were washed with staining buffer (PBS/2% FBS) and then incubated with FITC-conjugated anti-mouse CD80 and APC-conjugated anti-mouse CD206 for 15 min at room temperature. The expression of surface markers on the macrophages was measured with a flow cytometer via fluorescence detection. FlowJo software (Version 10.8)was applied to process the data collected from the flow cytometry experiments.

### 2.10. In Vitro Assessment of Cytokines Produced by Macrophages

To detect the levels of TNF-α (M1 macrophage-specific cytokine) and IL-10 (M2 macrophage-specific cytokine) after macrophages were treated with the MAPS, 1 × 10^5^ Raw 264.7 cells were seeded in 6-well plates and treated with the MAPS for 6 h, followed by NIR laser irradiation (0.75 W cm^−2^, 10 min) or not. Cells collected by trypsin were centrifuged for 10 min at 3000 rpm to remove dead cell or cell debris; then, the cytokines in the supernatant were assessed with an Enzyme Linked Immunosorbent Assay (ELISA) kit according to the manufacturer’s instruction.

### 2.11. In Vitro Antibacterial Activity Assessment

Direct photothermal antibacterial activity assessment: the antibacterial activity of the MAPS was evaluated with *Escherichia coli* (Gram-negative) and *Staphylococcus aureus* (Gram-positive) as the model bacteria: bacteria were grown on Luria–Bertani (LB) culture medium on a shaker under 150 rpm rotation at 37 °C overnight. Then, 600 μL diluted bacterial suspension (OD600 = 0.2) and 200 μL MAPS (the content of Mo was 100 μg mL^−1^) were mixed, followed by NIR laser irradiation (808 nm, 0.75 W cm^−2^, 10 min).

Synergistically, “direct photothermal burn” and “indirect killing by hot activated M1 macrophage” antibacterial activity assessments were conducted [43]: Raw 264.7 cells were inoculated into 6-well plates and washed with PBS 3 times after 24 h of culture; then, 600 μL diluted bacterial suspension (OD600 = 0.2) and 200 μL MAPS (the content of Mo was 100 μg mL^−1^) was added to the well, following by NIR laser irradiation (808 nm, 0.75 W cm^−2^, 10 min).

The bacterial suspension after the treatment was diluted 10,000 times and 50 μL was smeared on the LB plate. The agar plates were incubated at 37 °C for 24 h before the number of colonies was counted.

### 2.12. Cell Proliferation Assessment

Next, 2 × 10^4^ Raw 264.7 cells per well were seeded in the upper chambers of 24-well Transwell plates (Corning; polyester membrane; 6.5 mm diameter; 0.4 μm pore size), while 4 × 10^4^ 3T3 cells in 0.8 mL of DMEM medium were seeded into the lower chamber. After 24 h of incubation, the Raw 264.7 cells in the upper chamber were treated with a MAPS with a Mo content of 100 μg mL^−1^ for 24 h. After the treatments, the rate of 3T3 cell proliferation was determined following previous protocols [44,45]. Finally, fluorescent images were taken with a Nikon Ti-S fluorescence microscope. The fluorescence intensity of green/blue cells was analyzed with ImageJ software (Version 1.8.0).

### 2.13. In Vivo Wound Healing Effect

BALB/c female mice were purchased from Vital River Experiment Animal Technology Co., Ltd. (Beijing, China) with weight of around 18–20 g. The mice were divided into eight groups with three in each group (PBS/PBS + NIR, ACF/ACF + NIR, MoS_2_/MoS_2_ + NIR, MAPS/MAPS + NIR). The hair on the back of mice was removed with depilatory cream and then the skin was disinfected with alcohol cotton balls. A 1.2 cm transverse wound was cut on the dorsal skin of each mouse as a wound model. Then, 25 µL of MAPS (with a Mo content of 100 µg mL^−1^) was added to the wound, and the two sides of each wound edge were gently pressed for NIR laser irradiation (808 nm, 0.75 W cm^−2^, 10 min). The wounds were imaged and the length of the wounds was measured from Day 0 to Day 7. Wound length in each group was measured by ImageJ. The percentage of wound length on the indicated days in comparison with the original wound was calculated based on the following Equation (2):

(2) $${\mathrm{Woundlength}}_{\mathrm{day}\#}(\%)=\frac{{\mathrm{Woundlength}}_{\mathrm{day}\#}}{{\mathrm{Woundlength}}_{\mathrm{day}0}}\times 100\%$$

Wound length _day0_ and Wound length _day#_ represent the wound length after treatment at Day 0 and Day #, respectively.

The mice were sacrificed 7 days after the treatment, and tissue samples—including the wound area and the surrounding skin—were embedded in paraffin and cut into slices. The tissue samples were stained with hematoxylin and eosin (H&E) for FITC-conjugated anti-mouse CD80 and APC-conjugated anti-mouse CD206 immunofluorescence staining. Finally, the imaging of the stained sections was undertaken with a Nikon Ti-S fluorescence microscope.

The inflammatory cytokines TNF-α/IL-10 expressed in the wound area and the surrounding skin were analyzed using ELISA kits [46]. The tissue samples were centrifuged at 3000 rpm for 10 min. The concentrations of TNF-α and IL-10 in the supernatant from the various treatment groups were examined according to the manufacturer’s instructions.

The procedures involving experimental animals were in accordance with protocols approved by the Committee (2020018) for Animal Research of Changchun Institute of Applied Chemistry, Chinese Academy of Sciences, China.

### 2.14. Statistical Analysis

All data were tested at least three times. The mean± standard deviation is used to represent the final results, and *t*-tests were used to analyze the statistical significance between the two groups. * *p* < 0.05 was considered to be statistically significant.

## 3. Results

### 3.1. Physicochemical Structure Characterization of MAPS

**Figure 1:**
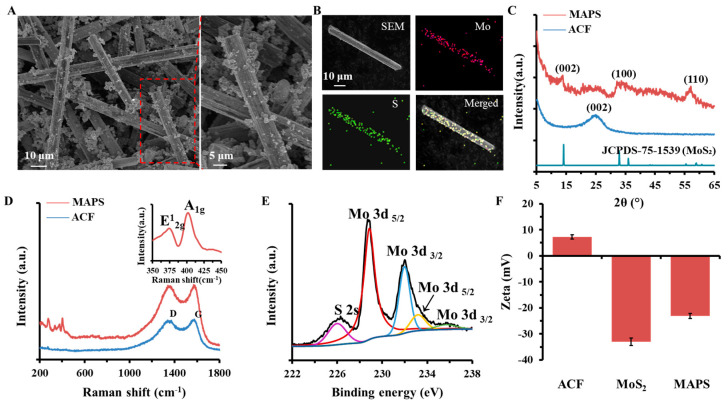
Physicochemical structure characterizations of MoS_2_, ACF, and MAPS. (**A**) SEM image of MAPS; (**B**) EDS mapping images of MAPS; (**C**) XRD patterns of ACF and MAPS; (**D**) Raman spectra of ACF and MAPS (insert: Raman spectra of MoS_2_); (**E**) XPS analysis of MAPS; (**F**) zeta potentials of MoS_2_, ACF and MAPS in aqueous solution (*n* = 3).

The MAPS was prepared via a hydrothermal method, as described in the Materials and Methods [39]. Scanning electron microscope (SEM) images indicated that the MoS_2_ was successfully incorporated into the ACF architecture, providing a rough surface (Figure 1A). The ACF kept its typically interconnected network structure with an average diameter and length of 7.03 ± 0.78 μm and 70.68 ± 18.57 μm, respectively. After being uniformly and densely anchored onto the ACF matrix, 440 nm was determined as the appropriate size for MoS_2_, and the average size of the MAPS was 1138 nm. Energy-dispersive X-ray spectroscopy (EDS) elemental mapping analysis revealed the homogeneous distributions of Mo and S contents across both sides of the fiber section, respectively (Figure 1B). The inductively coupled plasma optical emission spectroscopy (ICP-OES) results revealed that the percentage of the Mo element was 30 ± 5%. The XRD patterns suggest that the MAPS exhibited characteristic diffraction peaks of carbon and MoS_2_ (JCPDS No. 75-1539) (Figure 1C). The Raman spectra of both the ACF and the MAPS exhibited two distinct peaks at 1360.47 and 1584.32 cm^−1^ (Figure 1D), both corresponding to the D-band and G-band of carbon materials, respectively [40], and the characteristic peaks at 378.87 and 403.76 cm^−1^ corresponded to the in-plane E^1^_2g_ and the out-of-plane A_1g_ of MoS_2_ (illustration). X-ray photoelectron spectroscopy (XPS) revealed that the binding energies of Mo 3d_5/2_ and Mo 3d_3/2_ in the MAPS were 228.8 and 231.9 eV, respectively (Figure 1E), representing the existence of Mo^4+^, while the peaks located at 233.1 and 235.9 eV were attributed to Mo^6+^ [47]. The existence of both Mo^4+^ and Mo^6+^ indicates the potential antioxidant activity of MoS_2_ coupled with ACF [37]. The zeta potentials of MoS_2_, the ACF, and the MAPS were 7.2 ± 0.8, −33.0 ± 1.4 and −23.1 ± 1.0 mV, respectively (Figure 1F).

### 3.2. Photothermal Performance of MAPS

**Figure 2:**
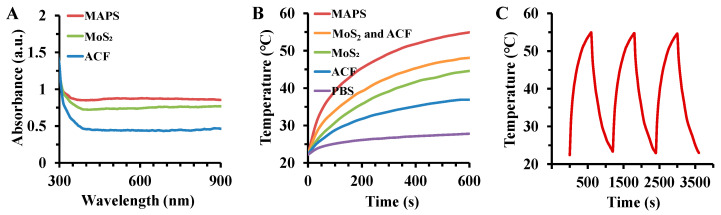
Photothermal performance of MoS_2_, ACF, and MAPS. (**A**) UV-VIS-NIR spectra of MoS_2_, ACF, and MAPS. (**B**) Temperature elevation profiles of MoS_2_, ACF, MAPS, and the MoS_2_ and ACF physical mixture suspended in PBS buffer under 808 nm laser irradiation (0.75 W cm^−2^, 10 min, MoS_2_ or ACF = 100 μg mL^−1^). (**C**) Thermal cycle stability of MAPS (0.75 W cm^−2^, 10 min, MoS_2_ or ACF = 100 μg mL^−1^).

Due to the strong absorption in the NIR region (Figure 2A), the MAPS showed concentration-dependent and power-density-dependent photothermal behavior upon NIR laser irradiation (Figure S1A,B). As shown in Figure 2B (808 nm laser irradiation, 0.75 W cm^−2^, 10 min), the MAPS exhibited excellent photothermal performance, which was superior to that of MoS_2_, ACF, and a physical mixture of MoS_2_ and ACF (equivalent to 100 μg mL^−1^ MoS_2_ or ACF). This result indicates that a strong electron transfer process occurs between MoS_2_ and ACF, which intensely depends on the construction of a MoS_2_-ACF heterojunction structure rather than a physical mixture [30,31]. The photothermal conversion efficiencies of the MAPS, MoS_2_, ACF, and the MoS_2_ and ACF physical mixture were calculated as 66.30, 39.59, 25.57, and 48.87%, respectively (Figure S2), indicating the better photothermal performance of the MAPS than the MoS_2_ and ACF physical mixture. After three radiation cycles, the MAPS did not show a noticeable change in photothermal performance, demonstrating its excellent photostability (Figure 2C) and further confirming the formation of a MoS_2_-ACF heterostructure.

### 3.3. Antioxidant Enzyme-Mimicking Activity of MAPS

**Figure 3:**
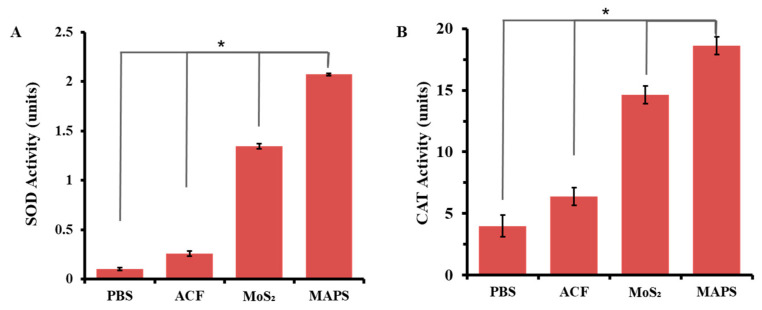
Antioxidant enzymatic activity of MAPS. (**A**) SOD activity; (**B**) CAT activity (MoS_2_ or ACF = 100 μg mL^−1^, *n* = 3, * *p* < 0.05).

Owing to its superoxide dismutase (SOD) and catalase (CAT) mimetic activity, MoS_2_ can act as an antioxidant to scavenge ROS, including hydrogen peroxide (H_2_O_2_) and superoxide (O_2_^−^), protecting cells and tissues from oxidative damage [48]. SOD catalyzes the dismutation of O_2_^−^ to H_2_O_2_, which is further detoxified to form O_2_ and H_2_O by CAT. The SOD-like and CAT-like activities of MoS_2_, ACF, and MAPS were detected by SOD and CAT assay kits, respectively. As shown in Figure 3, MoS_2_ and MAPS exhibited superoxide anion inhibition and H_2_O_2_ elimination ability due to the redox cycling between Mo^4+^ and Mo^6+^ [49]. Owing to the low conductivity of MoS_2_, electron transfer from Mo^4+^ to Mo^6+^ may be seriously blocked [50,51]. Coupling MoS_2_ with highly conductive AFC networks (with electrical conductivity of 4.3 × 10^3^ S m^−1^) provided an efficient pathway for the electron transfer from Mo^4+^ to Mo^6+^ (the electrical conductivity of the MAPS was 5.2 × 10^3^ S m^−1^). The superoxide anion inhibition rate and H_2_O_2_ elimination rate of the MAPS gradually increased as its concentration increased (Figure S3). Specifically, the steady-state kinetic analysis demonstrated that MAPSs with a lower Km value had a higher affinity for O_2_^−^ and H_2_O_2_ than MoS_2_ itself (Figure S4). All results confirmed that the formation of the MoS_2_-ACF heterostructure promoted antioxidant SOD and CAT activity, demonstrating promising ROS scavenging potential.

### 3.4. In Vitro Promotion of Inflammatory Processing by MAPS-Treated Macrophages with NIR Laser Irradiation

Considering biomedical applications, the cytotoxicity of the MAPS was evaluated in 3T3 cells with or without NIR laser irradiation under different MAPS concentrations, power densities, and irradiation times (Figure S5). Notably, the 100 μg mL^−1^ MAPS exhibited excellent biocompatibility with the 808 nm laser at 0.75 W cm^−2^ for 10 min.

**Figure 4:**
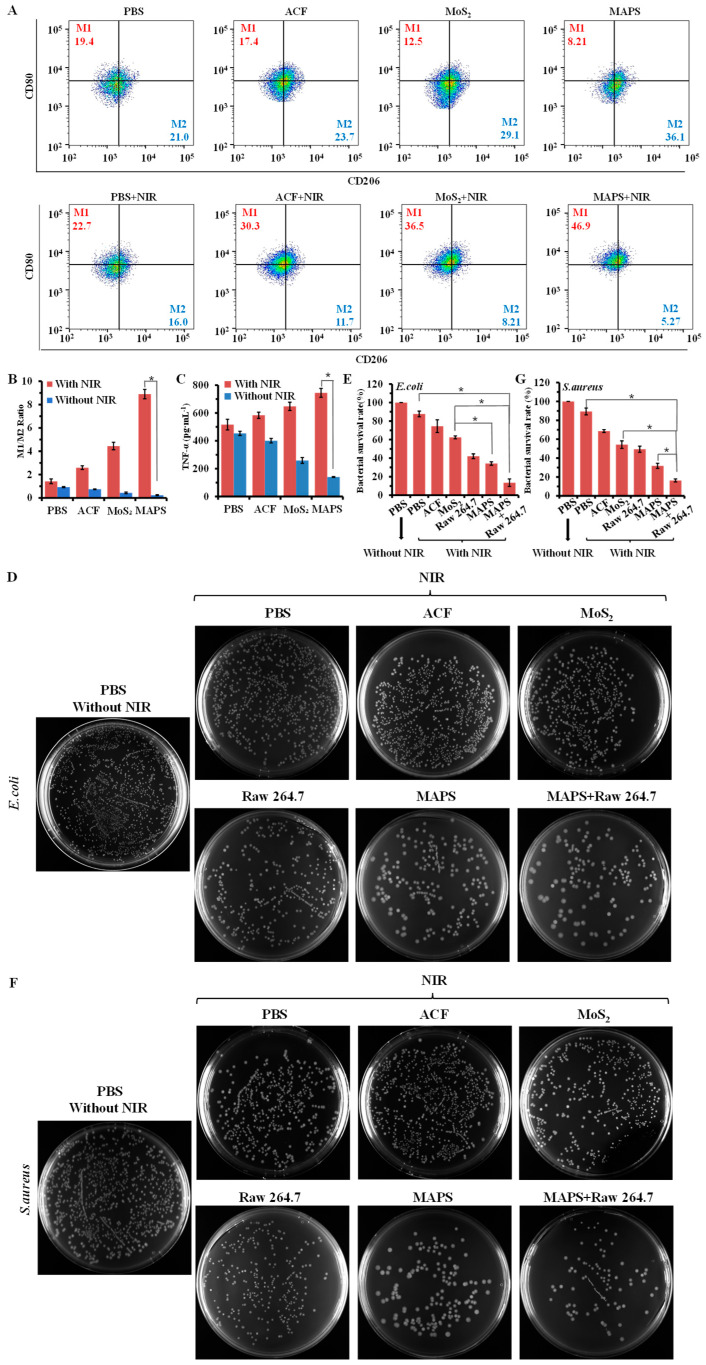
MAPS promotes macrophage reprogramming towards the pro-inflammatory M1 phenotype and inhibits bacteria growth with NIR laser irradiation. (**A**) Flow cytometry analysis of Raw 264.7 cells treated with PBS, ACF, MoS_2_, and MAPS, followed by 808 nm laser irradiation (0.75 W cm^−2^, 10 min, MoS_2_ or C = 100 μg mL^−1^) or not. (**B**) The ratios of M1/M2 in each group of (**A**). (**C**) The level of TNF-α release from Raw 264.7 cells treated with PBS, ACF, MoS_2_, and MAPS, followed by 808 nm laser irradiation (0.75 W cm^−2^, 10 min, MoS_2_ or ACF = 100 μg mL^−1^) or not. (**D**) Optical images of *Escherichia coli* colonies treated with PBS, ACF, MoS_2_, MAPS, Raw 264.7 and MAPS + Raw 264.7—followed by 808 nm laser irradiation (0.75 W cm^−2^, 10 min, MoS_2_ or ACF = 100 μg mL^−1^). (**E**) Bacterial survival analysis according to (**D**). (**F**) Optical images of *Staphylococcus aureus* colonies treated with PBS, ACF, MoS_2_, MAPS, Raw 264.7 and MAPS + Raw 264.7—followed by 808 nm laser irradiation (0.75 W cm^−2^, 10 min, MoS_2_ or ACF = 100 μg mL^−1^). (**G**) Bacterial survival analysis according to (**F**) (*n* = 3, * *p* < 0.05).

Mediated by the photothermal agent of the MAPS (Figure 2B), NIR laser irradiation could modulate the transformation of macrophages to the M1 phenotype, promoting inflammatory processing. Different characteristic surface protein markers are expressed by pro-inflammatory M1 macrophages and anti-inflammatory M2 macrophages. In this study, we used CD80 and CD206 as markers of M1 and M2 macrophages [52,53], respectively, to analyze the type of macrophage produced with different treatments. The M1 macrophages induced by the MAPS under 808 nm laser irradiation were evaluated by flow cytometry: the percentage of M1 macrophages (CD80+CD206−) (46.9%) was significantly enhanced compared with the control group (22.7%) (Figure 4A), and the ratio of M1/M2 macrophages increased from 1.4 to 8.9 (Figure 4B), indicating that the MAPS-potentiated photothermal property triggered the reprogramming of macrophages into the M1 phenotype [8,54]. As shown in Figure 4C, pro-inflammatory factor TNF-α (M1 marker) was released from the macrophages to clear the necrotic tissue, bacteria, and other harmful organisms at the wound site, demonstrating strong phagocytic ability [55].

Almost all open skin wounds contain bacteria, and the interaction between bacteria and the host might cause local infection, which would increase tissue damage, inhibit keratinocyte migration, disrupt the normal inflammation–proliferation phase transition, and finally lead to chronic inflammation and scarring [56,57]. In order to study the antibacterial effects of the MAPS, the colony-forming abilities of Gram-negative *Escherichia coli* (*E. coli*) and Gram-positive *Staphylococcus aureus* (*S. aureus*) were detected on an LB agar plate with or without NIR laser irradiation. Compared to the PBS-treated control, ACF-, MoS_2_-, and MAPS-treated bacteria showed little reduction in colony number (Figure S6). Under 808 nm laser irradiation, bacteria growth was suppressed when they were co-cultured with the MAPS: the survival rates were 34.20% and 31.82% for *Escherichia coli* and *Staphylococcus aureus*, respectively (Figure 4D–G), and both were lower compared with bacteria cultured on PBS. The bacterial elimination effect might be ascribed to MAPS-potentiated hyperthermia under NIR laser irradiation [55].

It is well known that macrophages play a pivotal role in the anti-infection reaction of the human body [4,7]. Since the photothermal effects of MAPSs could promote macrophages to switch toward the M1 phenotype (Figure 4A), we further evaluated the antibacterial ability of MAPSs co-cultured with M1 macrophages, synergistically combining “indirect killing by hot activated M1 macrophage” with “direct photothermal burn”. Under NIR laser irradiation, MAPS-treated macrophages more effectively inhibited bacterial growth than MAPSs alone, where the survival rate was only approximately 13.49% and 16.34% for *Escherichia coli* and *Staphylococcus aureus*, respectively (Figure 4D–G). These results demonstrate that the photothermal effects of MAPSs could not only directly kill bacteria, but also indirectly kill bacteria through inducing the reprogramming of macrophages toward M1.

### 3.5. In Vitro Modulation of Wound Healing Phase Transition by MAPS

**Figure 5:**
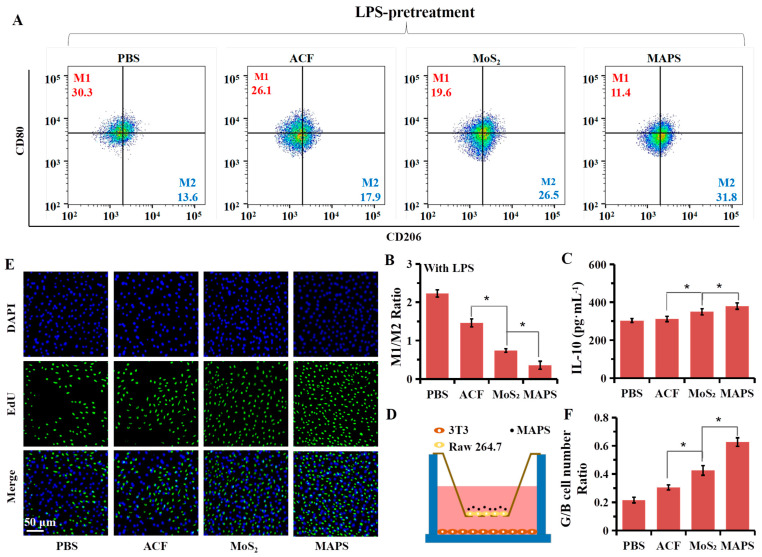
MAPS promotes macrophage phenotype switching to the anti-inflammatory M2 phenotype and accelerates cell proliferation. (**A**) Flow cytometry analysis of LPS-pretreated Raw 264.7 cells treated with PBS, ACF, MoS_2_, and MAPS (MoS_2_ or ACF = 100 μg mL^−1^). (**B**) The ratio of M1/M2 in each group of (**A**). (**C**) The levels of IL-10 release from LPS-pretreated Raw 264.7 cells treated with PBS, ACF, MoS_2_, and MAPS (MoS_2_ or ACF = 100 μg mL^−1^). (**D**) Schematic diagram of Raw 264.7/3T3 Transwell system. (**E**) Fluorescence image of 3T3 cells in Raw 264.7/3T3 Transwell system cultivated with MAPS (MoS_2_ or ACF = 100 μg mL^−1^) for 24 h and stained by EdU agent to evaluate cell proliferation. (**F**) Quantitative assessment of cell proliferation by ImageJ based on the green (G)/blue (B) cell number ratio in (**E**) (*n* = 3, * *p* < 0.05).

The MAPS itself can induce normal macrophages to transition to the M2 phenotype (Figure 4A,B and Figure S7). Moreover, for LPS-pretreated M1 macrophages, the MAPS decreased the proportion of the M1 phenotype from 30.3% to 11.4% and increased the M2 phenotype from 13.6% to 31.8% (Figure 5A), and the ratio of M1/M2 was reduced to only 0.36 (Figure 5B). The results imply that the MAPS alone can switch the phenotype of macrophages from M1 into M2, which is a result of its ROS scavenging properties (Figure 3) [22,23,24,25]. The released associated cytokine secretion of IL-10 (Figure 5C) from M2 macrophages could promote the transition from the inflammatory phase to the proliferative phase during wound healing, in line with the major theme of focusing on cell proliferation [55].

The acceleration of cell proliferation by the MAPS was studied in a Raw 264.7/3T3 Transwell system using an EdU assay (Figure 5D). 3T3 cells treated with MAPS and MoS_2_ showed more EdU-positive cells than cells treated with PBS. The enhancement of cell proliferation by the MAPS was more efficient than that by MoS_2_. Based on fluorescence image analysis, cells treated with the MAPS exhibited a higher green/blue cell number ratio than those with MoS_2_ (Figure 5E), which is attributed to the higher antioxidant activity of the MAPS than MoS_2_ alone (Figure 3) [15,22]. These results suggest that MAPS-treated macrophages can significantly promote cell proliferation because the MAPS can efficiently switch macrophages into the M2 phenotype via its antioxidant activity.

### 3.6. In Vivo Wound Healing by MAPS

**Figure 6:**
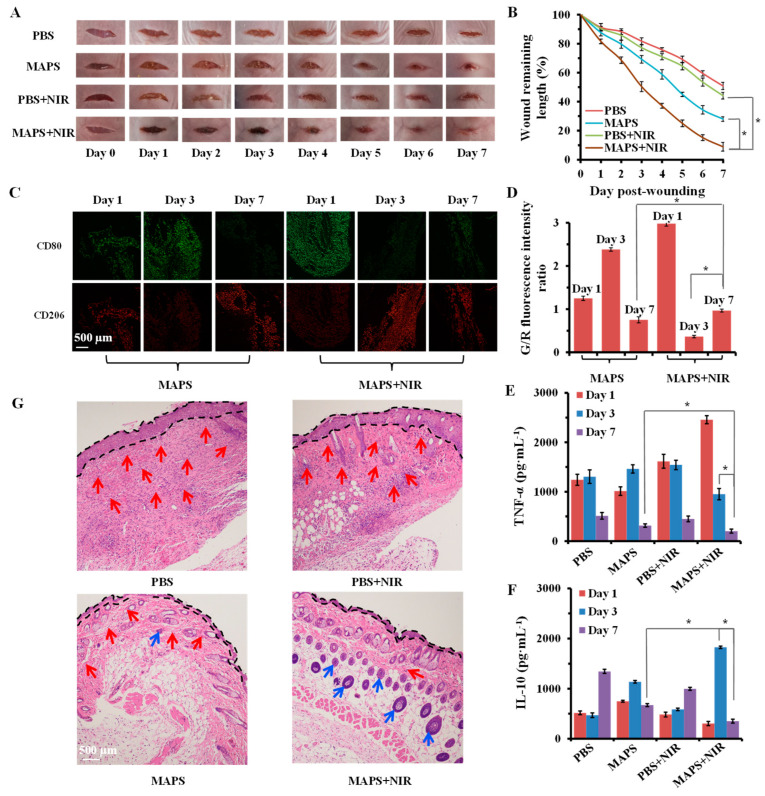
In vitro wound healing performance of MAPS. (**A**) Wound repair images over 7 days of treatments with or without NIR laser irradiation (0.75 W cm^−2^, 10 min). (**B**) Quantification of wound repair kinetics expressed as percentage of the initial wound length. (**C**) Fluorescence staining images of CD 80 (green) and CD 206 (red) in wound tissue after treatment. (**D**) Fluorescence intensity ratio analysis according to (**C**). The levels of TNF-α (**E**) and IL-10 (**F**) in wound area after treatment (detected by ELISA). (**G**) H&E-stained wound tissue images at Day 7 post-wounding: the black dashed lines show the boundary of the epidermal layer, red arrows indicate inflammatory cells, and the blue arrows indicate new hair follicles (*n* = 3, * *p* < 0.05).

The transition from inflammation to proliferation may be one of the most critical and fateful steps in wound healing. On the basis that the excellent modulation of M1/M2 macrophage phenotypes can initiate the resolution of inflammation and trigger proliferation, the MAPS may be beneficial for accelerating wound healing in vivo. A cut wound was established on the back of mice as a common main wound model to evaluate the healing effect of the MAPS in an acute wound model. According to the pictures of the wounds (Figure 6A and Figure S8), after coating the MAPS suspension in PBS at the wound beds, NIR laser irradiation could accelerate wound healing. The shrinkage of wound size in the MAPS-treated group was quicker than that in the other groups: 7 days after the MAPS and NIR laser treatments, the average wound length remaining was about 8.93%, smaller than that of the PBS-treated group (50.83%) and MAPS-treated group without NIR laser irradiation (28.03%) (Figure 6B). Additionally, the wound was repaired with a smooth appearance, and a less irregular ragged reddish scar was found with both the MAPS and NIR laser treatments (Figure 6A and Figure S8, Day 7), demonstrating that the MAPS could condense the inflammatory phase under NIR laser irradiation, therefore prolonging the proliferative period.

The inflammatory phase lasts 3 days in a normal wound healing process; then, the transition from inflammation to proliferation occurs (Figure 6C,D). To explore the mechanisms by which the MAPS modulated the wound microenvironment, wound tissues after different treatments were collected on Day 1, 3, and 7. As shown in Figure 6C, there were a great number of M1 macrophages (CD80+) in the wound tissue after the MAPS and NIR laser treatment, demonstrating that the MAPS induced macrophages to a pro-inflammatory M1 phenotype with NIR laser irradiation and rapidly switched the macrophages to the reparative M2 phenotype (CD206+) after the NIR laser was removed.

To further investigate the effective wound phase transition with the MAPS and NIR laser treatment, the levels of several inflammation-associated cytokines in wound tissues were quantified by ELISA (Figure 6E,F, Figure S9 and Figure S10). The contents of TNF-α were much higher in the MAPS + NIR laser group, suggesting that the resident M1 macrophages were activated and the inflammatory response could be promptly amplified on Day 1 (Figure 6E). At the later stage of the healing process, the MAPS yielded a more eminent ROS scavenging effect than MoS_2_, demonstrating that the macrophages transited from the pro-inflammatory M1 phenotype to the reparative M2 phenotype, regulating the wound healing transition from inflammation to proliferation. Anti-inflammatory mediator cytokines of IL-10 (Figure 6F) released from M2 macrophages could enhance cell proliferation. In the mouse model, the wound was cured 7 days after the MAPS and NIR laser treatment.

After mice were sacrificed, the wound tissue sections were stained with H&E to evaluate the internal structure and condition of the healed wound (Figure 6G and Figure S11). Compared with the control groups, after the MAPS and NIR laser treatment, the wound epidermis was almost completely connected, no wound gaps were observed, and the scar width was significantly reduced. All of the above results indicate that the MAPS could efficiently regulate the inflammation–proliferation transition, promote wound healing, and minimize scar formation.

## 4. Conclusions

Macrophage M1-M2 transition is critical for the resolution of inflammation and the initiation of proliferation. In the present study, we successfully constructed a MoS_2_-ACF heterojunction structure as a macrophage M1-M2 phenotype switcher (MAPS) for regulating the inflammation–proliferation transition to accelerate wound healing. Coupling MoS_2_ with ACF endowed the MAPS with excellent photothermal and antioxidative performance in terms of enhanced electron transfer. Under 808 nm laser irradiation, MAPS-potentiated hyperthermia not only killed the bacterial directly, but also activated pro-inflammatory M1 macrophages to indirectly kill bacteria and control inflammation to minimize scar formation. Then, owing to its remarkable antioxidant activity, the MAPS could induce macrophage switching towards the M2 phenotype to resolve inflammation and promote cell proliferation. Overall, our work provides a promising therapeutic approach to wounds to re-set the wound immuno-environment and optimize tissue regeneration. In the future, to develop new wound healing materials, some important elements should be considered: tunable mechanical properties should be included in the materials to match the surrounding tissue and promote wound healing; stimuli-responsive materials should be developed for the precise control of wound healing; and various growth factors should be released from materials for angiogenesis, cellular proliferation, and tissue regeneration.

## Data Availability

The original contributions presented in the study are included in the article, further inquiries can be directed to the corresponding author.

## Supplementary Materials

The following supporting information can be downloaded at: https://www.mdpi.com/article/10.3390/ma18050963/s1, Figure S1. Comparison of temperature elevation profiles of MAPS at different concentrations under NIR laser irradiation (0.75 W cm^−2^, 10 min) (A) and different NIR laser power (MoS_2_ = 100 μg mL^−1^) (B). Figure S2. The monitored temperature changing curves of MoS_2_, ACF, MAPS and physical mixture of MoS_2_&ACF as irradiated by the NIR laser (0.75 W cm^−2^, 10 min, MoS_2_ or ACF = 100 μg mL^−1^), followed by natural cooling with the laser light turned off, and determination of the time constant for heat transfer from the system using linear regression of the cooling profiles. (A) Temperature variation curves; (B) Linear regression curves. Figure S3. Superoxide anions inhibition (A) and H_2_O_2_ scavenging (B) by MAPS in a dose-dependent manner. Figure S4. Steady-state kinetic assay and catalytic mechanism of MoS_2_ and MAPS. Michaelis-Menten and corresponding Lineweaver-Burk plot (insert) with various concentrations of (A) Xanthine and (B) H_2_O_2_. Figure S5. Comparisons of 3T3 cells viability after treated with different concentrations of MAPS (according to Mo content) for 6 h, followed by 808 nm laser irradiation with different power density or for different irradiation time. Figure S6. Optical images of bacterial colonies formed by *E. coli* (A) and *S. aureus* (B) treated with PBS, ACF, MoS_2_ and MAPS, Raw 264.7 and MAPS + Raw 264.7 (MoS_2_ or ACF = 100 μg mL^−1^) without NIR laser irradiation; (C) *E. coli* survival analysis according to (A); (D) *S. aureus* survival analysis according to (B). (mean ± SD, t-test, *n* = 3, * *p* < 0.05). Figure S7. The levels of IL-10 release from Raw 264.7 treated with PBS, ACF, MoS_2_ and MAPS (MoS_2_ or ACF = 100 μg mL^−1^). (mean ± SD, t-test, *n* = 3, * *p* < 0.05). Figure S8. In vivo wound healing performance of ACF or MoS_2_. Wound repair images during 7 days of treatments with or without NIR laser irradiation (0.75 W cm^−2^, 10 min); Quantification of wound repair kinetics expressed as percentage of the initial wound length. (mean ± SD, t-test, *n* = 3, * *p* < 0.05). Figure S9. The values of TNF-α in wound area after treatment (detected by ELISA). (mean ± SD, t-test, *n* = 3, * *p* < 0.05). Figure S10. The values of IL-10 in wound area after treatment (detected by ELISA). (mean ± SD, *t*-test, *n* = 3, * *p* < 0.05). Figure S11. H&E-stained wound tissue images at Day 7 postwounding: the black dashed lines show the boundary of the epidermal layer, red arrows indicate inflammatory cells, and the blue arrows indicate new hair follicles.
