# Supplementary material for: Construction of Antibacterial MoS2-ACF Phenotype Switcher for Bidirectionally Regulating Inflammation–Proliferation Transition in Wound Healing
Source: Materials (Basel). 2025 Feb 21;18(5):963. doi: 10.3390/ma18050963 (PMC11901119; doi:10.3390/ma18050963)
Supplement: Supplementary file 1 [file materials-18-00963-s001.zip › materials-3440635-supplementary.pdf]

Supplementary Materials

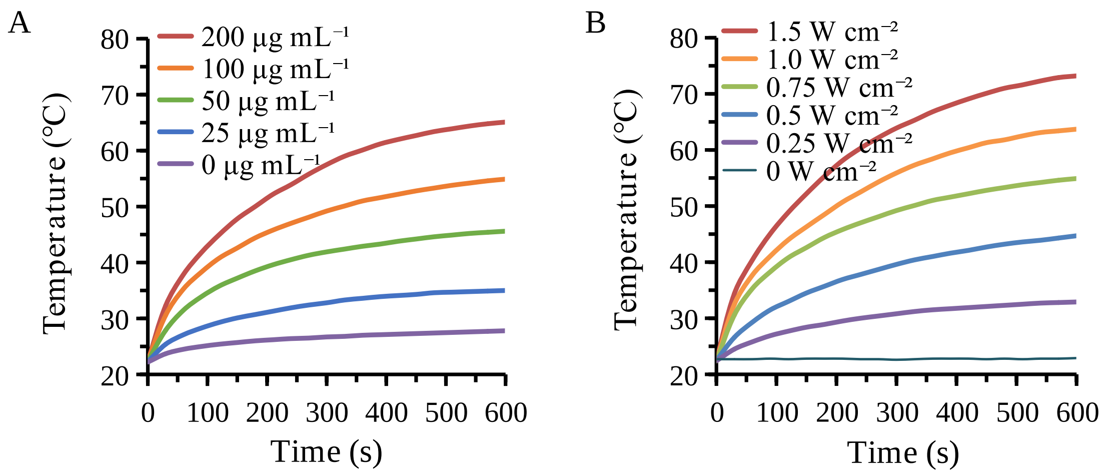

Figure S1. Comparison of temperature elevation profiles of MAPS at different concentrations under NIR laser irradiation (0.75 W cm^{-2}, 10 min) (A) and different NIR laser power (MoS_2=100 μg mL^{-1}) (B).

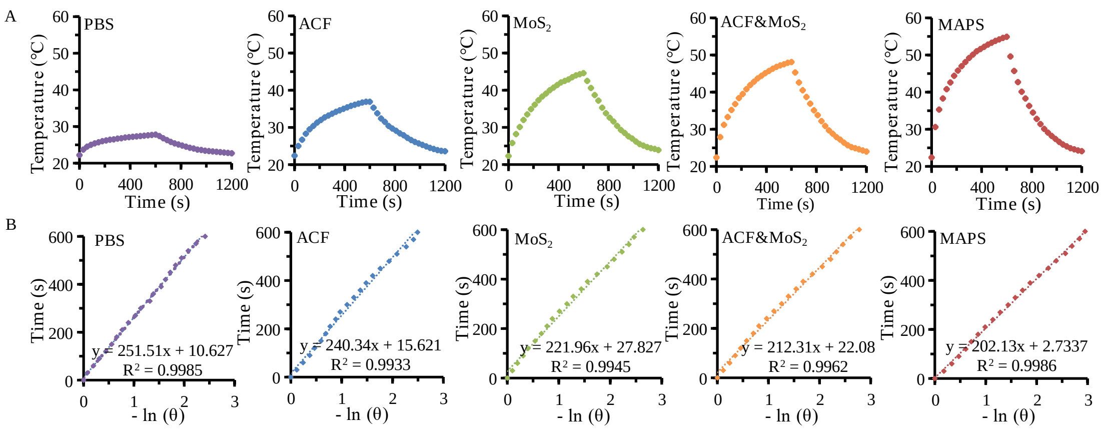

Figure S2. The monitored temperature changing curves of MoS_2, ACF, MAPS and physical mixture of MoS_2&ACF as irradiated by the NIR laser (0.75 W cm^{-2}, 10 min, MoS_2 or ACF =100 μg mL^{-1}), followed by natural cooling with the laser light turned off, and determination of the time constant for heat transfer from the system using linear regression of the cooling profiles. (A) Temperature variation curves; (B) Linear regression curves.

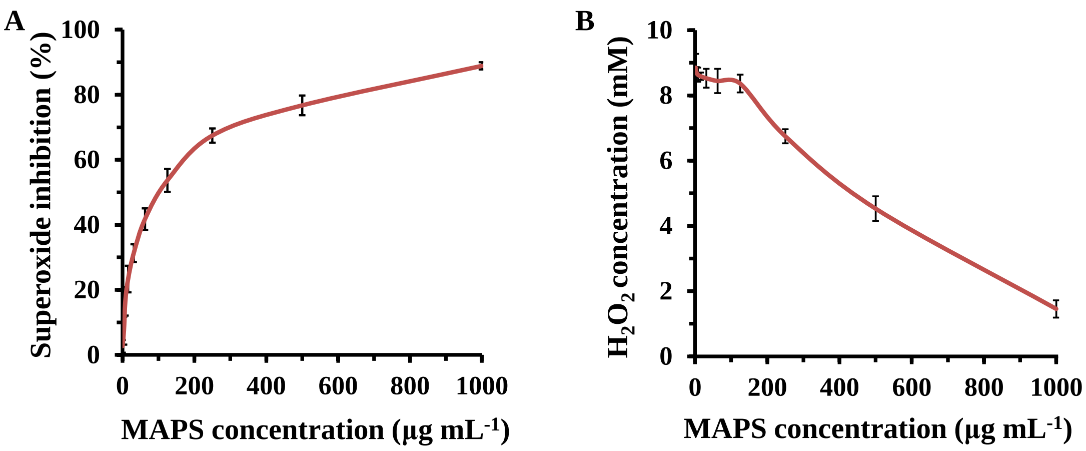

Figure S3. Superoxide anions inhibition (A) and H_2O_2 scavenging (B) by MAPS in a dose-dependent manner.

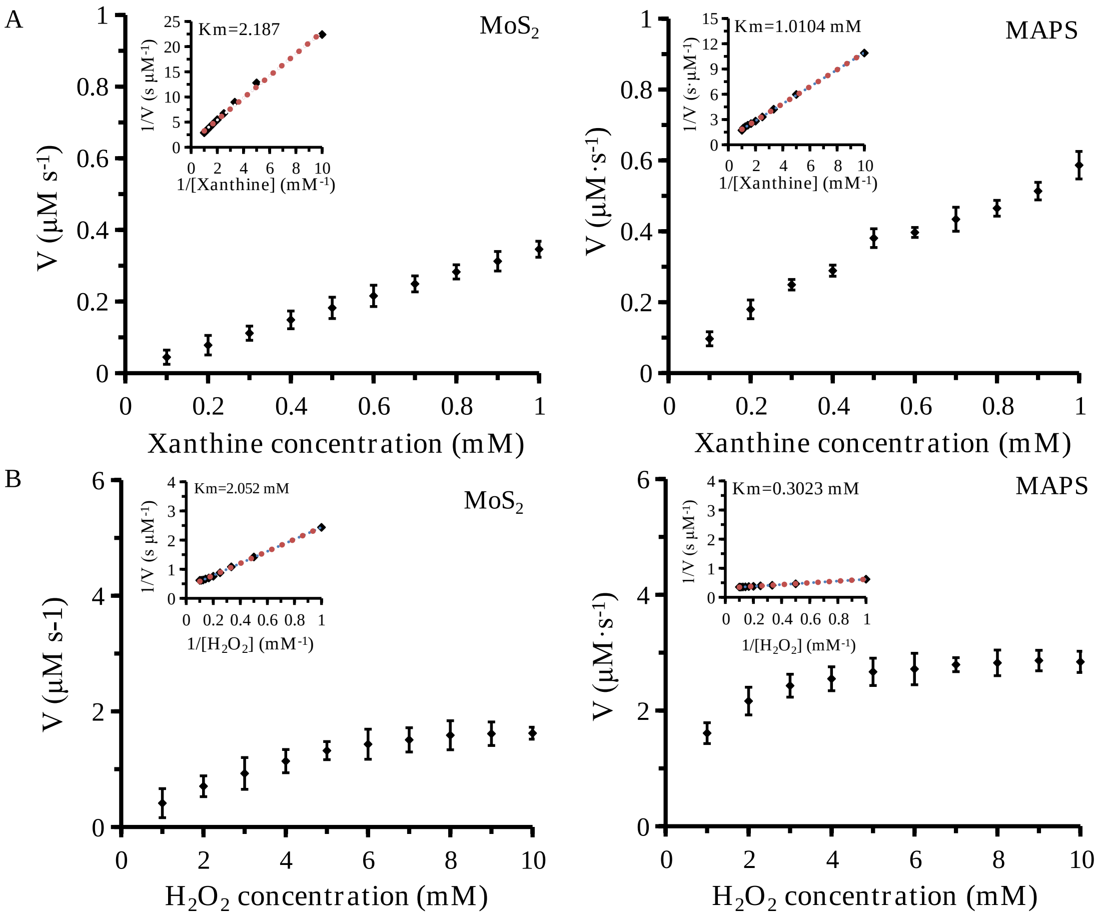

Figure S4. Steady-state kinetic assay and catalytic mechanism of MoS_2 and MAPS. Michaelis-Menten and corresponding Lineweaver-Burk plot (insert) with various concentrations of (A) Xanthine and (B) H_2O_2.

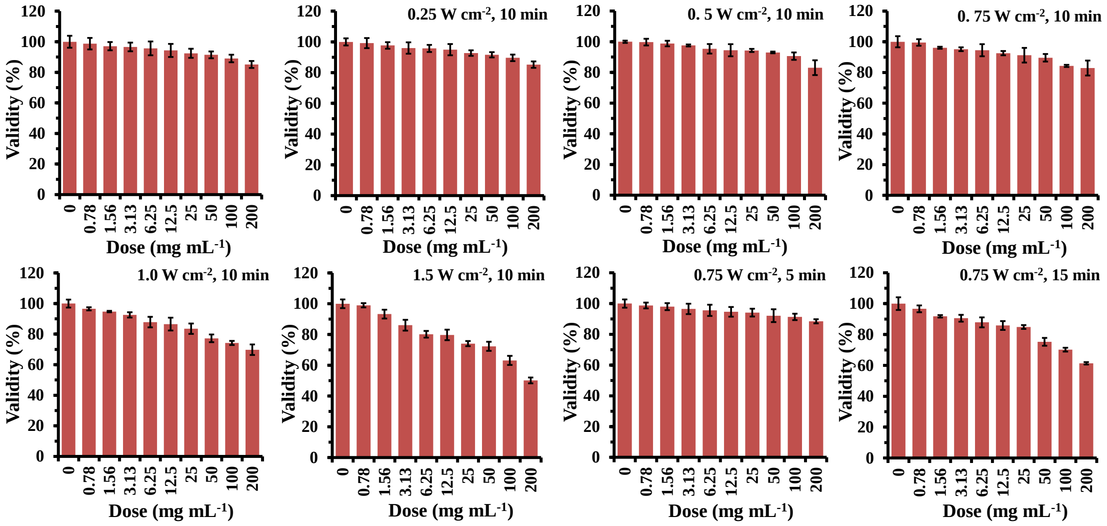

Figure S5. Comparisons of 3T3 cells viability after treated with different concentrations of MAPS (according to Mo content) for 6 h, followed by 808 nm laser irradiation with different power density or for different irradiation time.

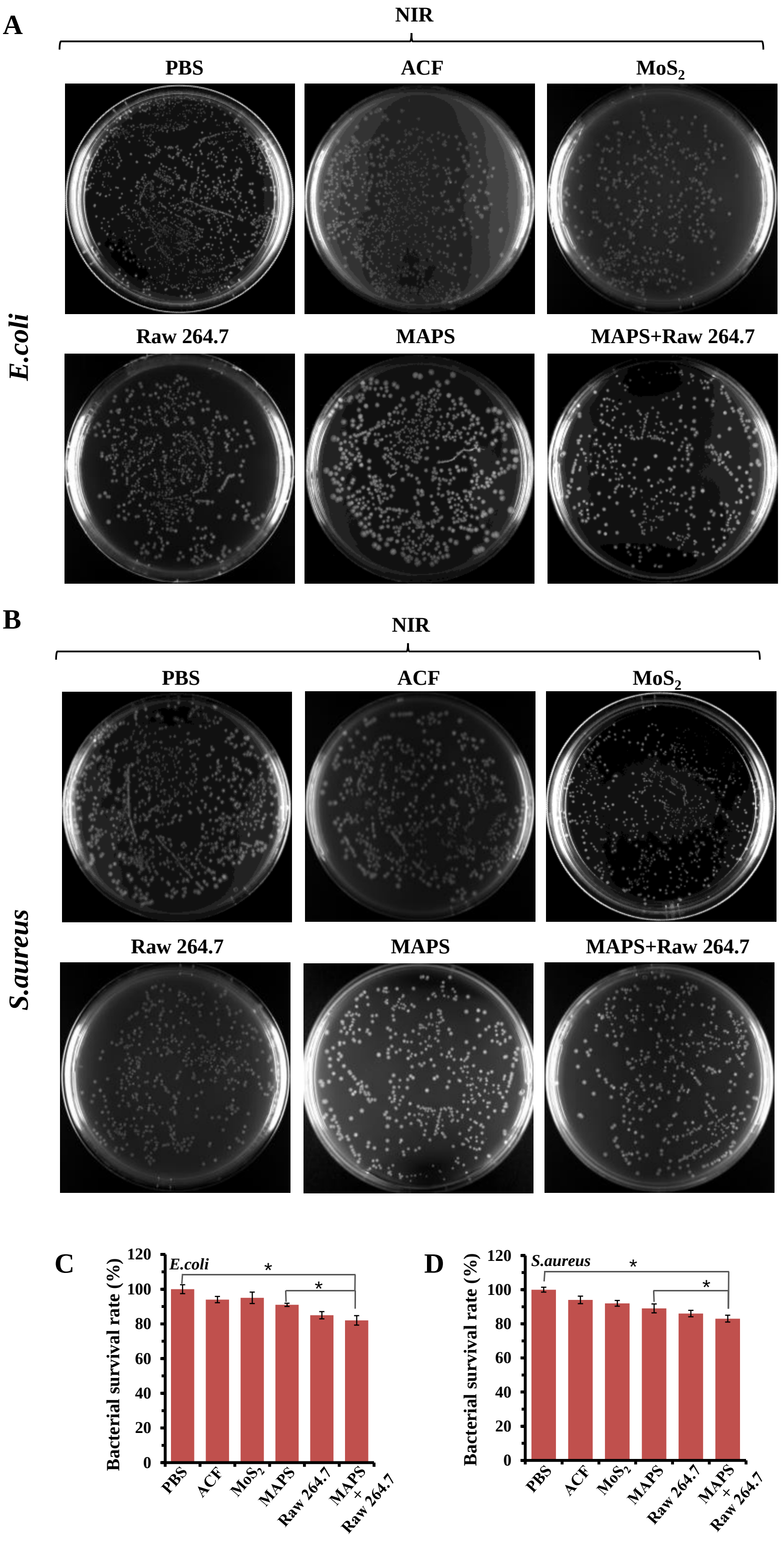

Figure S6. Optical images of bacterial colonies formed by *E. coli* (A) and *S. aureus* (B) treated with PBS, ACF, MoS_2 and MAPS, Raw 264.7 and MAPS+Raw 264.7 (MoS_2

or ACF =100 μg mL^{-1}) without NIR laser irradiation; (C) *E. coli* survival analysis according to (A); (D) *S. aureus* survival analysis according to (B). (mean ± SD, t-test, n=3, *P<0.05).

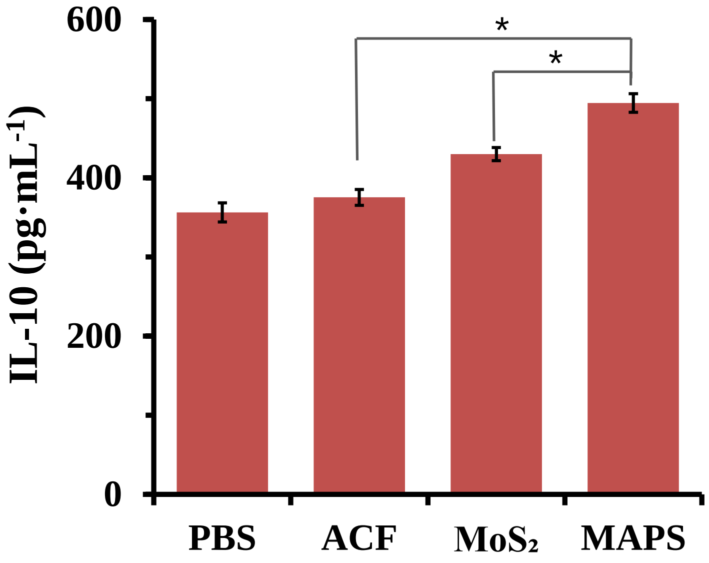

Figure S7. The levels of IL-10 release from Raw 264.7 treated with PBS, ACF, MoS_2 and MAPS (MoS_2 or ACF =100 μg mL^{-1}). (mean ± SD, t-test, n=3, *P<0.05).

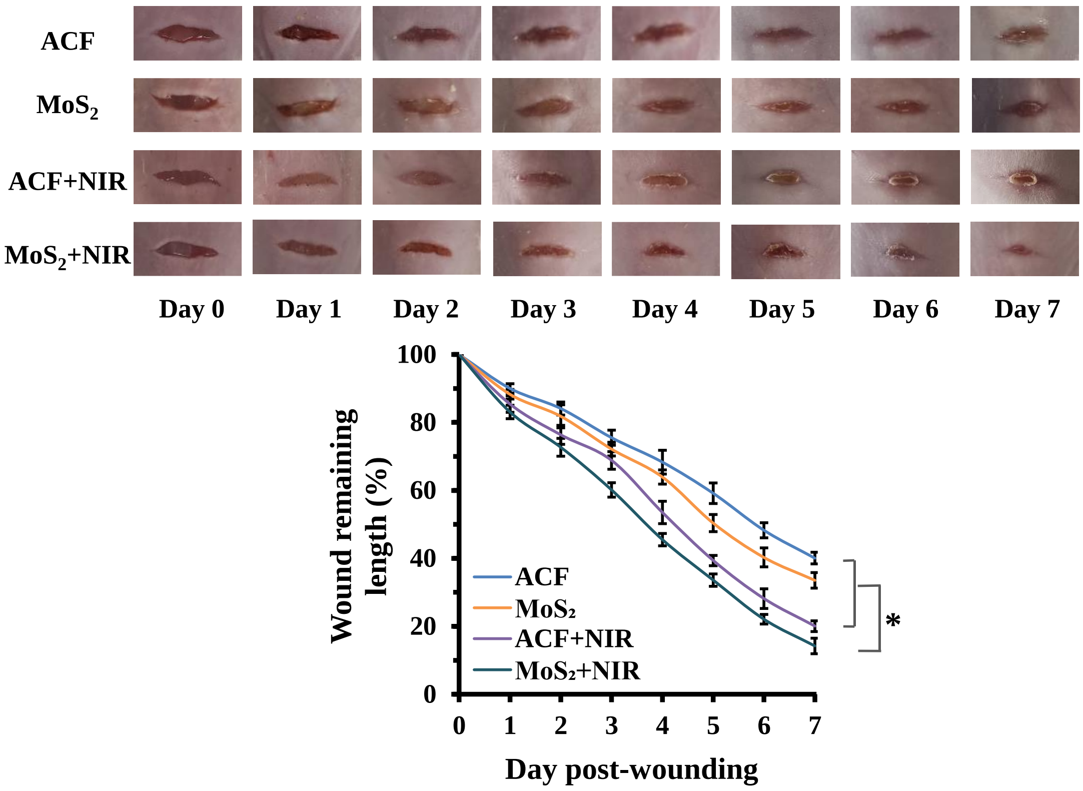

Figure S8. *In vivo* wound healing performance of ACF or MoS_2. Wound repair images during 7 days of treatments with or without NIR laser irradiation (0.75 W cm^{-2}, 10 min); Quantification of wound repair kinetics expressed as percentage of the initial wound length. (mean ± SD, t-test, n=3, *P<0.05).

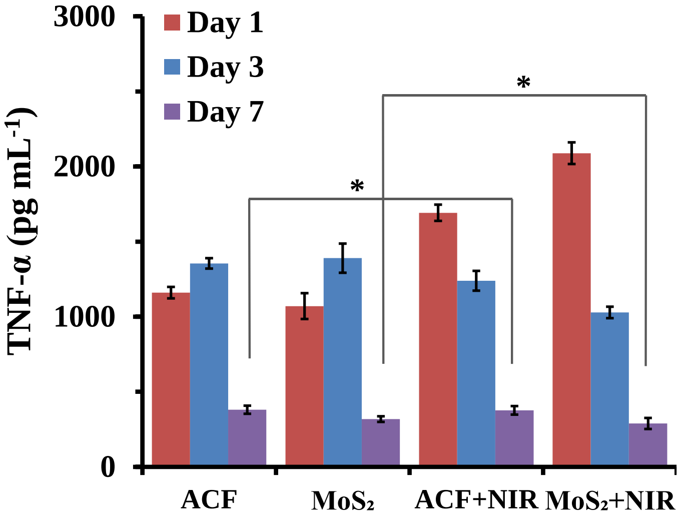

Figure S9. The values of TNF-α in wound area after treatment (detected by ELISA).

(mean ± SD, t-test, n=3, *P<0.05)

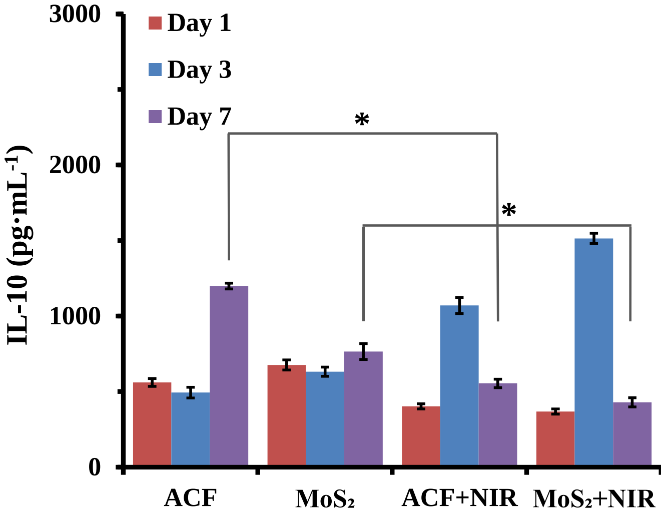

Figure S10. The values of IL-10 in wound area after treatment (detected by ELISA). (mean ± SD, t-test, n=3, *P<0.05).

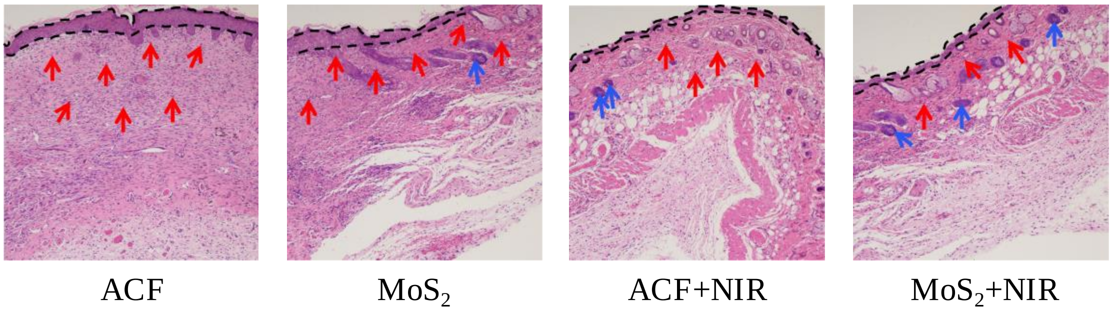

Figure S11. H&E-stained wound tissue images at Day 7 postwounding: the balck dashed lines show the boundary of the epidermal layer, red arrows indicate inflammatory cells, and the blue arrows indicate new hair follicles.
